# Supplementary figures and images for: Competition Sensing Changes Antibiotic Production in Streptomyces
Source: mBio. 2021 Feb 9;12(1):e02729-20. doi: 10.1128/mBio.02729-20 (PMC7885098; doi:10.1128/mBio.02729-20)

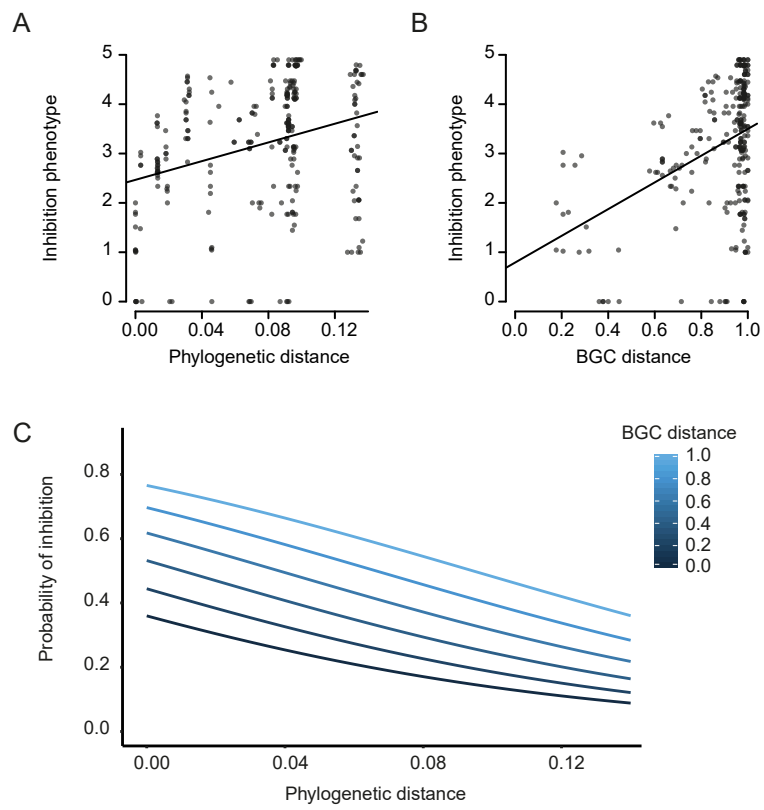

**Fig. S1**

Supplement: FIG S1 [file mBio.02729-20-sf001.pdf]

A

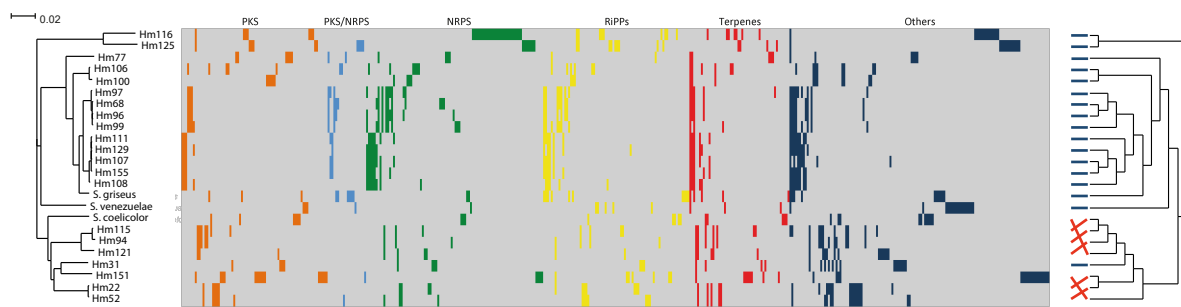

B

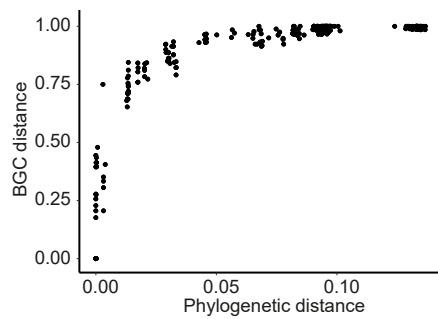

**Fig. S2**

Supplement: FIG S2 [file mBio.02729-20-sf002.pdf]

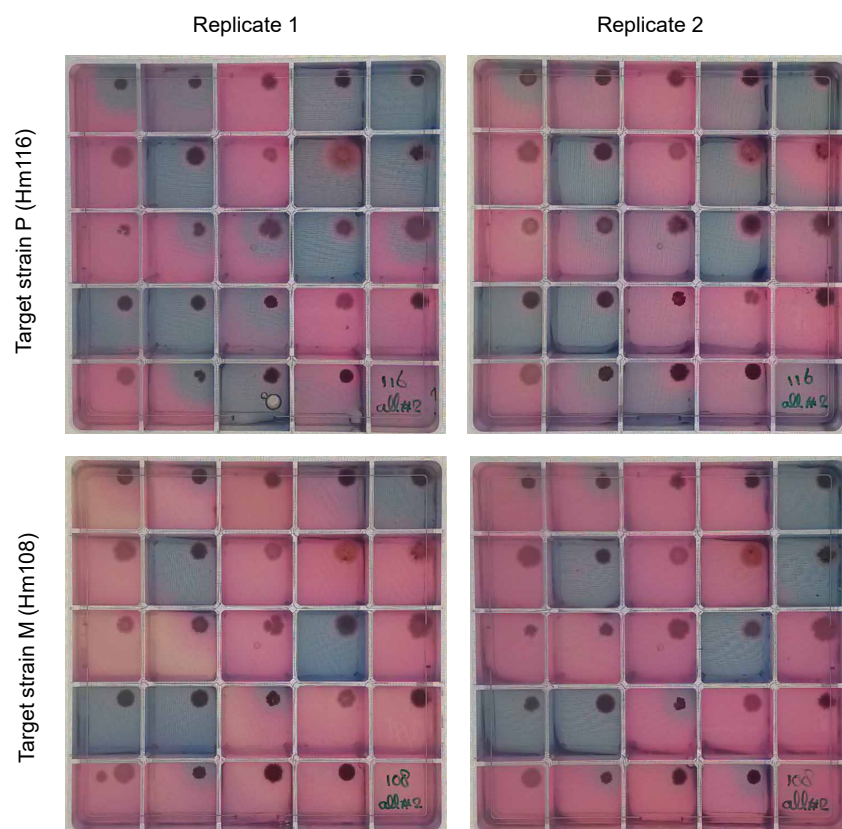

Focal strain

|   |   |   |   |   |
|---|---|---|---|---|
| L | N | F | J | H |
| B | D | P | T | R |
| V | X | A | E | C |
| G | I | U | O | W |
| Q | S | K | M |   |

**Fig. S4**

Supplement: FIG S4 [file mBio.02729-20-sf004.pdf]
