# Supplementary material for: Competition Sensing Changes Antibiotic Production in Streptomyces
Source: mBio. 2021 Feb 9;12(1):e02729-20. doi: 10.1128/mBio.02729-20 (PMC7885098; doi:10.1128/mBio.02729-20)
Supplement: TABLE S1 [file mBio.02729-20-st001.pdf]

Table S1. Strains used in this study. All strains designated 'Hm' were isolated from the same soil sample taken from the Himalaya (1). Most of these strains were included in the MBT set described in the same paper and can be found under the corresponding 'MBT' names in other publications.

Identifier	Strain name	Nr of BGC	Nr of scaffolds	N50
A	Hm22/MBT97	36	1146	14743
B	Hm31/MBT33	29	537	41669
C	Hm52/MBT49	37	764	24257
D	Hm68/MBT98	36	635	24564
E	Hm77/MBT51	25	357	41187
F	Hm94/MBT53	27	542	43101
G	Hm96/MBT54	33	498	30984
H	Hm97/MBT55	36	591	26110
I	Hm99/MBT56	34	589	27955
J	Hm100/MBT57	22	3232	4281
K	Hm106/MBT58	33	622	27814
L	Hm107/MBT59	31	596	25060
M	Hm108/MBT60	30	552	26977
N	Hm111/MBT61	30	598	25388
O	Hm115/MBT62	32	758	31686
P	Hm116/MBT63	64	1037	13653
Q	Hm121/MBT65	27	833	24761
R	Hm125/MBT66	42	44	714511
S	Hm129/MBT67	31	602	24967
T	Hm151/MBT70	55	395	69644
U	Hm155/MBT72	31	497	31206
V	*S. coelicolor* A3(2) M145	29		
W	*S. venezuelae* ATCC 15439	30		
X	*S. griseus* IFO 13350	36		

1. Zhu H, Swierstra J, Wu C, Girard G, Choi YH, van Wamel W, Sandiford SK, van Wezel GP. 2014. Eliciting antibiotics active against the ESKAPE pathogens in a collection of actinomycetes isolated from mountain soils. Microbiology 160:1714–1725.
