# Supplementary material for: Competition Sensing Changes Antibiotic Production in Streptomyces
Source: mBio. 2021 Feb 9;12(1):e02729-20. doi: 10.1128/mBio.02729-20 (PMC7885098; doi:10.1128/mBio.02729-20)
Supplement: FIG S3 [file mBio.02729-20-sf003.pdf]

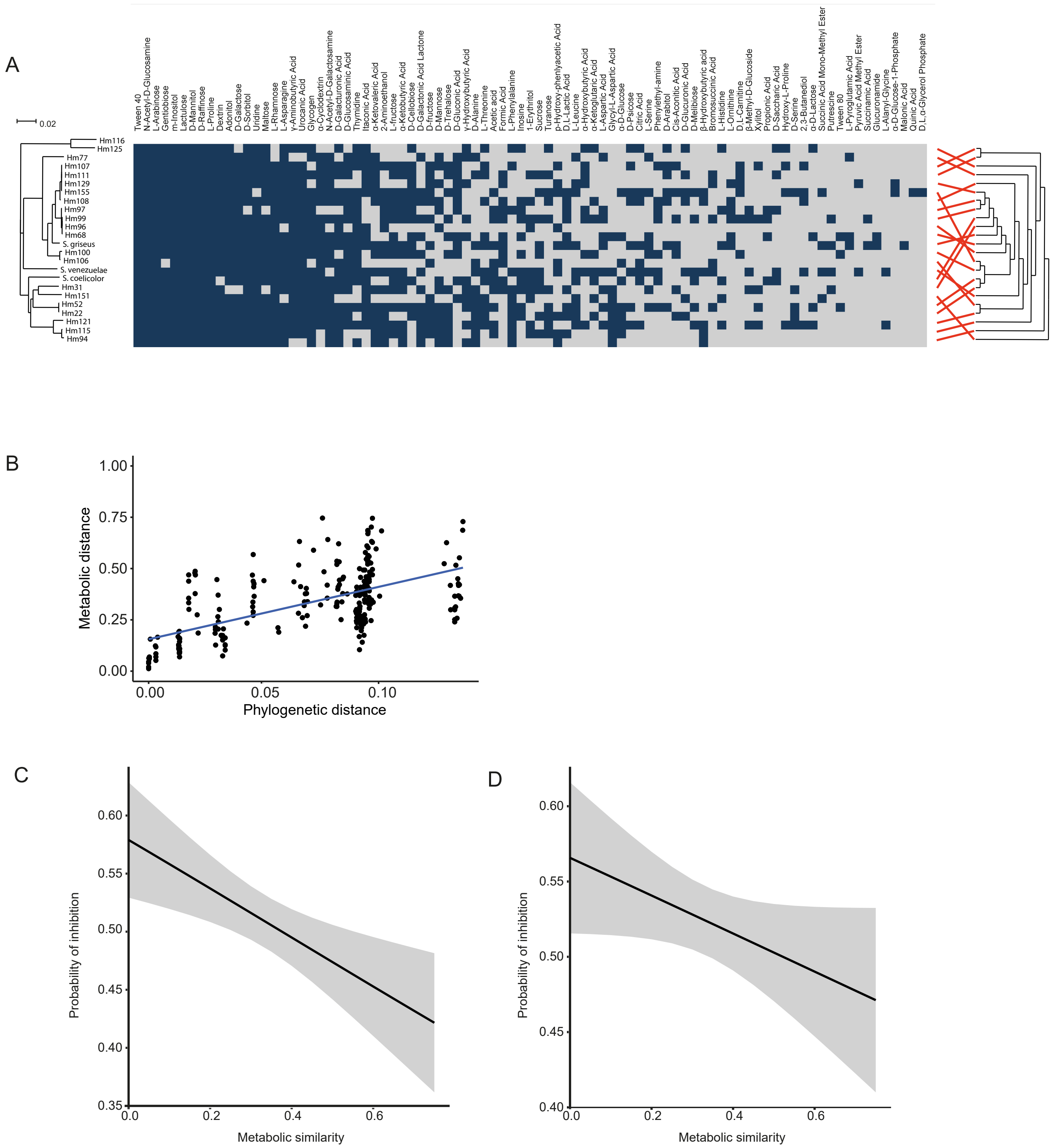
A
0.02
Hm116
Hm125
Hm77
Hm107
Hm111
Hm129
Hm155
Hm108
Hm97
Hm99
Hm96
Hm68
S. griseus
Hm100
Hm106
S. venezuelae
S. coelicolor
Hm31
Hm151
Hm52
Hm22
Hm121
Hm115
Hm94
B
Metabolic distance
1.00
0.75
0.50
0.25
0.00
0.00
0.05
0.10
Phylogenetic distance
C
Probability of inhibition
0.60
0.55
0.50
0.45
0.40
0.35
0.0
0.2
0.4
0.6
Metabolic similarity
D
Probability of inhibition
0.60
0.55
0.50
0.45
0.40
0.0
0.2
0.4
0.6
Metabolic similarity

Fig. S3
